# Association between maternal outdoor physical exercise and the risk of preterm birth: a case-control study in Wuhan, China

**DOI:** 10.1186/s12884-021-03678-9

**Published:** 2021-03-12

**Authors:** Miao Cai, Bin Zhang, Rong Yang, Tongzhang Zheng, Guanghui Dong, Hualiang Lin, Steven E. Rigdon, Hong Xian, Leslie Hinyard, Pamela K. Xaverius, Echu Liu, Thomas E. Burroughs, Daire R. Jansson, Morgan H. LeBaige, Shaoping Yang, Zhengmin Qian

**Affiliations:** 1 Department of Epidemiology, School of Public Health, Sun Yat-sen University, 74 Zhongshan 2nd Road, Yuexiu District, Guangzhou, 510080 China; 2 Wuhan Children’s Hospital (Wuhan Maternal and Child Healthcare Hospital), Tongji Medical College, Huazhong University of Science & Technology, 100 Xianggang Road, Jiangan District, Wuhan, 430015 China; 3 Department of Epidemiology Brown School of Public Health, 121 S Main St, Providence, RI 02903 USA; 4 Department of Toxicology, School of Public Health, Sun Yat-sen University, 74 Zhongshan 2nd Road, Yuexiu District, Guangzhou, 510080 China; 5 Department of Epidemiology and Biostatistics, College for Public Health & Social Justice, Saint Louis University, 3545 Lafayette Avenue, Saint Louis, MO 63104 USA; 6 Center for Health Outcomes Research, Saint Louis University, 3545 Lafayette Avenue, Saint Louis, MO 63104 USA; 7 Department of Health Management and Policy, College for Public Health and Social Justice, Saint Louis University, 3545 Lafayette Avenue, Saint Louis, MO 63104 USA

**Keywords:** Preterm birth, Physical exercise, Pregnancy, Bayesian, Nonlinear, Wuhan

## Abstract

**Background:**

China had the second largest proportion of preterm birth (PTB) internationally. However, only 11% of pregnant women in China meet international guidelines for maternal physical activity, a significantly lower proportion than that in Western countries. This study aims to examine the association between outdoor physical exercise during pregnancy and PTB among Chinese women in Wuhan, China.

**Methods:**

A case-control study was conducted among 6656 pregnant women (2393 cases and 4263 controls) in Wuhan, China from June 2011 to June 2013. Self-reported measures of maternal physical exercise (frequency per week and per day in minutes) were collected. Adjusted odds ratios were estimated using Bayesian hierarchical logistic regression and a generalized additive mixed model (GAMM).

**Results:**

Compared to women not involved in any physical activity, those who participated in physical exercise 1–2 times, 3–4 times, and over five times per week had 20% (aOR: 0.80, 95% credible interval [95% CI]: 0.68–0.92), 30% (aOR: 0.70, 95% CI: 0.60–0.82), and 32% (aOR: 0.68, 95% CI: 0.59–0.78) lower odds of PTB, respectively. The Bayesian GAMM showed that increasing physical exercise per day was associated with lower risk of PTB when exercise was less than 150 min per day; however, this direction of association is reversed when physical exercise was more than 150 min per day.

**Conclusion:**

Maternal physical exercise, at a moderate amount and intensity, is associated with lower PTB risk. More data from pregnant women with high participation in physical exercise are needed to confirm the reported U-shape association between the physical exercise and risk of preterm birth.

**Supplementary Information:**

The online version contains supplementary material available at 10.1186/s12884-021-03678-9.

## Introduction

Preterm births (PTB), defined as births that occurred at a gestational age of less than 37 weeks, accounted for approximately 35% of deaths among newborn babies globally in 2016 [1–3]. Even though over 50% of preterm neonates survive, they face substantially higher risks of short-term and long-term morbidity and mortality [4]. Common complications of PTB, including respiratory distress syndrome, bronchopulmonary dysplasia, and sepsis, were the leading causes of death in children less than five years old in 2016 [3]. PTB poses a significant threat to healthcare, social security, and the economy, but few evidence-based strategies have been developed to effectively reduce the incidence of PTB [1–3, 5].

It is generally recommended that pregnant women maintain an adequate level of physical exercise. The United States Department of Health and Human Services advises pregnant women to participate in at least two and a half hours of aerobic activity per week with moderate-intensity [6]. The British Nutrition Foundation suggests that being physically active is beneficial to both the pregnant woman and the baby [7]. Health departments in different countries also provided similar recommendations [8]. Although being physically active is recommended for pregnant women to maintain overall good health, these guidelines do not explicitly claim that physical activity is directly associated with a decreased risk of PTB.

Biologically, physical exercise produces higher levels of adrenaline, noradrenaline, and catecholamines, which may induce preterm birth via myometrial contractions and intrauterine growth restriction, or induce myometrial relaxation according to the action of receptors such as alpha 1, 2 and beta 1, 2 receptors [8–10]. However, physical exercise can improve placental vascularization and decrease oxidative stress in pregnant women, both of which may reduce the risk of PTB [9, 11]. Empirically, several randomized controlled trials (RCTs) and observational studies have reported that aerobic exercise was associated with a reduction in the incidence of PTB [8, 12–15]. A systematic review reported that aerobic exercise of 30 to 90 min three to four times a week is not associated with increased risk of preterm birth, while the study simultaneously did not claim that physical exercise is associated with reduced risk of preterm birth [10]. Other studies reported a null association between physical activity and the risk of preterm birth [16, 17]. In summary, there is not a clear consensus in theoretical mechanisms or scientific research on the association between maternal physical activity and PTB risk.

It is estimated China had the second largest proportion of PTB internationally in 2014, second only to India [3, 18]. In Hubei province in which this study was conducted, the incidence of preterm birth has reportedly increased rapidly from 56.7 in 2001 to 105.2 in 2012 per 1000 live births [19]. Additionally, only 11% of pregnant women in China meet the international guidelines for physical activity during pregnancy, a significantly lower proportion than that in Western countries [20]. Understanding the association between physical exercise and PTB may help reduce the prevalence of PTB and create substantial value for maternal health in China. Utilizing matched-case control survey data from preterm and full-term births in Wuhan, China from June 2011 to June 2013, this study aims to examine the association between maternal physical activity and PTB and to evaluate the potential dose-response shape of the relationship.

## Methods

### Data sources

The study is a case-control study nested in a population-based birth cohort in Wuhan, China [21, 22]. Wuhan is the largest city in central China and serves as a transportation hub. There are approximately 10 million people in Wuhan, and over 60% of them reside in seven core, urban districts: Jiangan, Jianghaan, Qiaokou, Hanyang, Wuchang, Qingshan, and Hongshan.

The cases and controls were sampled from the Electronic Perinatal Health Care Information System (EPHCIS) in Wuhan Medical and Health Center for Women and Children from June 9, 2011 to June 10, 2013 [22–25]. The EPHCIS collects clinical data including antepartum examination, intra-partum condition, as well as post-partum visit data for both the mother and neonates. The participants were required to meet the following inclusion criteria: (1) have lived in the seven core urban districts for at least 2 years; (2) gave birth in a hospital located in the seven core urban districts; (3) did not have a record of mental disorder prior to pregnancy; (4) gave birth to a newborn with a gestational age of 28 to 42 weeks and weight of 500 to 5000 g; and (5) had a singleton live birth without birth defects.

The participants were sampled three times a month: on the 10th, 20th, and the last day of each month. Births from the 1st to the 10th day of the month were sampled on the 20th day; births from the 11th to 20th days were sampled on the last day of the month; births after the 20th day were sampled on the 10th day of the following month. Controls were matched to cases based on birth date within a 10-day window to account for potential effects of weather and environmental exposures [22–25]. In total, 6656 pregnant women agreed to participate in the study: 2393 PTB cases and 4263 full-term birth controls. All study participants provided written informed consent prior to data collection and were administered a questionnaire by trained interviewers. To minimize the risk of recall bias, all the participants were required to complete their questionnaire within 42 days after the baby was born. This study was approved by the Institutional Review Board of Saint Louis University (approval number 16744). Study reporting followed the Strengthening the Reporting of Observational Studies in Epidemiology (STROBE) reporting guideline.

### Outcome and exposure measurement

The outcome, PTB, was dichotomized as full-term birth (37 to 42 weeks of gestation) and PTB (less than 37 completed weeks of gestation) [22–25]. Gestation age was calculated as the date since the last date of menstrual period if the participant (93.7%) could accurately provide that date; otherwise, it was estimated by ultrasound scan performed in the gestational age of 24 to 26 weeks (6.3%) [23].

We measured maternal physical activity using two questions: “How many times did you exercise regularly, for 30 to 40 minutes per week, during the late gestational period (after seven gestational months)?” and “How much time did you spend on outdoor sports per day during your pregnancy?” The first question created a five-level variable, including “none” (reference group), “1 to 2 times”, “3 to 4 times”, “more than 5 times”, and “a doctor or nurse advised you not to exercise”. The second question produced a continuous variable that measured the number of minutes the women spent participating in outdoor sports per day during their pregnancy.

### Covariates

The covariates in this study were selected based on risk factors reported in previous literature and data availability [3, 18, 26, 27]. Maternal age (less than or equal to 21, 22–28, and > 28 years of age), education (less than high school, high school or occupational school, college, and graduate school), and household income (1000–2999 RMB as low, 3000–6999 RMB as middle, and more than 6999 RMB as high income, with 1 USD equaling around 6.5 RMB at the time of this study) were included as socioeconomic variables. Maternal parity was dichotomized as first baby or at least one prior live birth. Family history of premature or low birth weight newborns and newborn gender were also included. Since the prevalence of each of the following maternal comorbidities was all less than 1%, we created a dichotomous variable for selected maternal comorbidity, with one indicating that the mother has at least one of the following comorbidities: diabetes, thyroid disease, hepatitis, hypertension, heart disease, renal disease, epilepsy, asthma, depression, anxiety, or others [28, 29]. Anemia and colporrhagia were included as separate comorbidities since they have higher prevalence (more than 10%). Since only 35 (0.5%) participants were obese prior to pregnancy, we combined “overweight” and “obese”; therefore, body mass index prior to pregnancy was classified as underweight (BMI < = 18.5), normal weight (18.5–24.9), and overweight/obese (> = 25). Weight gain during pregnancy was collected in kilograms. Indoor air pollution was classified as no abnormal smells or noticeable smell (including tolerable odor, uncomfortable odor, and heavy odor) in the residence.

### Statistical analyses

Characteristics of the overall sample and by preterm status were reported as mean ± standard deviation for continuous variables and frequency (percent) for categorical variables. The differences between those with full-term and preterm birth were assessed using unpaired t-tests (for continuous variables) and Chi-square tests (for categorical variables).

For the five-level physical exercise variable (exercise frequency per week), we assessed the predictors of preterm birth using a Bayesian hierarchical logistic regression, with a random intercept for each of the seven core urban districts to account for potential geographic clustering. Since this is a matched case-control study where cases and controls were loosely matched by the date of birth, we conducted a conditional logistic regression model with the same predictors as a sensitivity test for any potential estimation bias caused by the case-control study design.

To detect any possible nonlinear relationships, we conducted a Bayesian generalized additive mixed model (GAMM) with random intercepts for the seven inner-city districts [30]. The number of basis functions was set at 10 to capture any potential nonlinear relationship, and the smoothing parameters were selected using the package’s default optimizing algorithm [31, 32]. We also conducted a similar model using different knot setting to detect any significant change due to spline specification as a sensitivity analysis. We did not perform a conditional GAMM as a sensitivity test since no applicable software package could perform that model. As recommended by the package developers, we assigned weakly informative priors to the parameters as follows: Normal (0, 10^2^) for intercept parameters, Normal (0, 5^2^) for all non-intercept parameters, and Gamma (1, 1) for scale (standard deviation) parameters. To ensure that the Markov chain Monte Carlo (MCMC) algorithm converged, we set four chains with 2000 warm-up (burn-in) and 5000 iteration steps for each chain. We used Gelman-Rubin statistics [33], posterior trace plots, and the effective sample sizes as diagnostic statistics [34]. Adjusted odds ratios (aORs) and 95% credible intervals (CIs) were reported. All data cleaning, visualization, and statistical modeling were performed using the statistical computing environment R 4.0.3 [35].

## Results

### Characteristics of the overall study sample and by preterm status

The characteristics of the overall study sample and by preterm status are presented in Table 1. Compared to those who did not experience PTB, mothers who experienced PTB tended to participate in significantly less physical exercise during pregnancy, be younger, had newborns that were male, have more than one parity, have less weight gain and more maternal comorbidity. No statistically significant differences were found between cases and controls regarding education, income, BMI prior to pregnancy, prevalence of anemia, and indoor air pollution.

**Table 1:** Characteristics of the overall study sample and by preterm birth status

	Overall (***n*** = 6656)	Full-term (***n*** = 4263)	Preterm (***n*** = 2393)	***P***-values
Exercise per day, in minutes, mean ± SD	43.4 ± 24.3	44.0 ± 24.6	42.1 ± 23.6	0.012
Exercise frequency per week, no (%)				< 0.001
Zero	1881 (28.3)	1107 (26.0)	774 (32.3)	
1–2	1285 (19.3)	834 (19.6)	451 (18.8)	
3–4	1179 (17.7)	802 (18.8)	377 (15.8)	
> 5	2170 (32.6)	1476 (34.6)	694 (29.0)	
Doctors suggested not to	141 (2.1)	44 (1.0)	97 (4.1)	
Maternal age, no (%)				< 0.001
< 20 years	1148 (17.2)	618 (14.5)	530 (22.1)	
20 to < 25 years	2966 (44.6)	1902 (44.6)	1064 (44.5)	
25 to < 30 years	2213 (33.2)	1529 (35.9)	684 (28.6)	
≥ 30 years	329 (4.9)	214 (5.0)	115 (4.8)	
Education, no (%)				0.112
Less than high school	1279 (19.2)	825 (19.4)	454 (19.0)	
High school or occupational school	1917 (28.8)	1262 (29.6)	655 (27.4)	
College	3131 (47.0)	1960 (46.0)	1171 (48.9)	
Graduate school	329 (4.9)	216 (5.1)	113 (4.7)	
Income, no (%)				0.058
Middle income (3000–6999 RMB)	4482 (67.3)	2910 (68.3)	1572 (65.7)	
Low income (< 3000 RMB)	626 (9.4)	379 (8.9)	247 (10.3)	
High income (> = 7000 RMB)	1548 (23.3)	974 (22.8)	574 (24.0)	
Family history, no (%)	297 (4.5)	129 (3.0)	168 (7.0)	< 0.001
Neonatal gender: male, no (%)	3699 (55.6)	2273 (53.3)	1426 (59.6)	< 0.001
Parity: more than one, no (%)	1119 (16.8)	682 (16.0)	437 (18.3)	0.02
BMI prior to pregnancy, no (%)				0.149
Underweight	1325 (19.9)	869 (20.4)	456 (19.1)	
Normal	4933 (74.1)	3154 (74.0)	1779 (74.3)	
Overweight/Obesity	398 (6.0)	240 (5.6)	158 (6.6)	
Weight gain in kilograms, mean ± SD	14.3 ± 4.0	14.5 ± 4.0	13.8 ± 3.8	< 0.001
Maternal comorbidity, no (%)	499 (7.5)	248 (5.8)	251 (10.5)	< 0.001
Anemia, no (%)	684 (10.3)	439 (10.3)	245 (10.2)	0.972
Colporrhagia, no (%)	1668 (25.1)	865 (20.3)	803 (33.6)	< 0.001
Indoor air pollution, no (%)	1060 (15.9)	671 (15.7)	389 (16.3)	0.605
District, no (%)				0.047
Hanyang	997 (15.0)	634 (14.9)	363 (15.2)	
Hongshan	967 (14.5)	640 (15.0)	327 (13.7)	
Jiangan	1256 (18.9)	760 (17.8)	496 (20.7)	
Jianghan	821 (12.3)	527 (12.4)	294 (12.3)	
Qiaokou	933 (14.0)	588 (13.8)	345 (14.4)	
Qingshan	510 (7.7)	337 (7.9)	173 (7.2)	
Wuchang	1172 (17.6)	777 (18.2)	395 (16.5)	

Maternal comorbidity includes any of the following comorbidities: diabetes, thyroid disease, hepatitis, hypertension, heart disease, renal disease, epilepsy, asthma, depression, anxiety, or others

*SD* standard deviation

### Association between physical exercise category per week and preterm birth

We observed significant and graded associations between the three physical exercise categories and PTB in the Bayesian hierarchical logistic regression (Table 2). Compared to mothers who never participated in physical exercise, those who participated in physical exercise 1 to 2 times a week (aOR: 0.80, 95% CI: 0.68–0.92), 3 to 4 times a week (aOR: 0.70, 95% CI: 0.60–0.82), and more than 5 times a week (aOR: 0.68, 95% CI: 0.59–0.78) all had considerably lower chances of experiencing PTB. The mothers who were advised against physical exercise by their doctors or nurses (aOR: 2.50, 95% CI: 1.70–3.70) had significantly higher chances of experiencing PTB compared to those who did not conduct any physical exercise. To account for the nature of the matched case-control study design, we also performed a conditional logistic regression as a sensitivity analysis (Table 2). The parameter estimates for physical exercise in the conditional logistic model were consistent with those estimated using the Bayesian unconditional logistic models.

**Table 2:** Adjusted odds ratios (95% credible intervals) for predictors of preterm birth

Characteristics	Hierarchical logistic regression	Conditional logistic regression
Physical exercise frequency per week (Reference: zero)		
1–2	0.80 (0.68–0.92)	0.85 (0.75–0.97)
3–4	0.70 (0.60–0.82)	0.80 (0.70–0.91)
≥ 5	0.68 (0.59–0.78)	0.80 (0.71–0.89)
Doctors suggested not to	2.50 (1.70–3.70)	1.53 (1.20–1.94)
Maternal age, years (Reference: 25 to < 30 years)		
< 20 years	1.68 (1.43–1.98)	1.38 (1.20–1.57)
20 to < 25 years	1.16 (1.02–1.31)	1.12 (1.01–1.25)
≥ 30 years	1.27 (0.97–1.65)	1.12 (0.91–1.39)
Education (Reference: less than high school)		
High school or occupational school	0.97 (0.83–1.14)	0.98 (0.86–1.12)
College	1.14 (0.97–1.35)	1.06 (0.93–1.21)
Graduate school	0.97 (0.72–1.28)	0.92 (0.73–1.17)
Income (Reference: middle income, 3000–6999 RMB)		
Low income (< 3000 RMB)	1.22 (1.01–1.46)	1.15 (0.99–1.33)
High income (≥7000 RMB)	1.05 (0.93–1.20)	1.02 (0.91–1.13)
Family history	2.28 (1.78–2.91)	1.48 (1.25–1.76)
Neonatal gender: male	1.28 (1.16–1.43)	1.16 (1.06–1.27)
Parity: more than one	0.96 (0.82–1.13)	0.97 (0.85–1.10)
BMI prior to pregnancy (Reference: normal weight)		
Underweight	1.03 (0.90–1.17)	1.00 (0.90–1.12)
Overweight/Obesity	1.00 (0.80–1.25)	0.98 (0.82–1.17)
Weight gain (per one kilogram increase)	0.96 (0.94–0.97)	0.97 (0.96–0.98)
Maternal comorbidity	1.71 (1.41–2.07)	1.33 (1.15–1.54)
Anemia	0.97 (0.81–1.15)	0.98 (0.85–1.13)
Colporrhagia	1.87 (1.66–2.10)	1.43 (1.30–1.57)
Indoor air pollution	0.97 (0.84–1.11)	0.99 (0.88–1.11)

Maternal comorbidity includes any of the following comorbidities: diabetes, thyroid disease, hepatitis, hypertension, heart disease, renal disease, epilepsy, asthma, depression, anxiety, or others

Parameter estimates of the two Bayesian hierarchical models had over 3000 effective sample sizes in MCMC draws, and their associated Gelman-Rubin statistics were all less than 1.1. The four chains were well-mixed without any divergent chains or significant serial-correlation (Supplemental Figure 1A). All evidence indicated that the MCMC algorithms converged and that the estimates were valid and trustworthy.

### Nonlinear relationship between physical exercise per day and risk of preterm birth

**Fig. 1:**
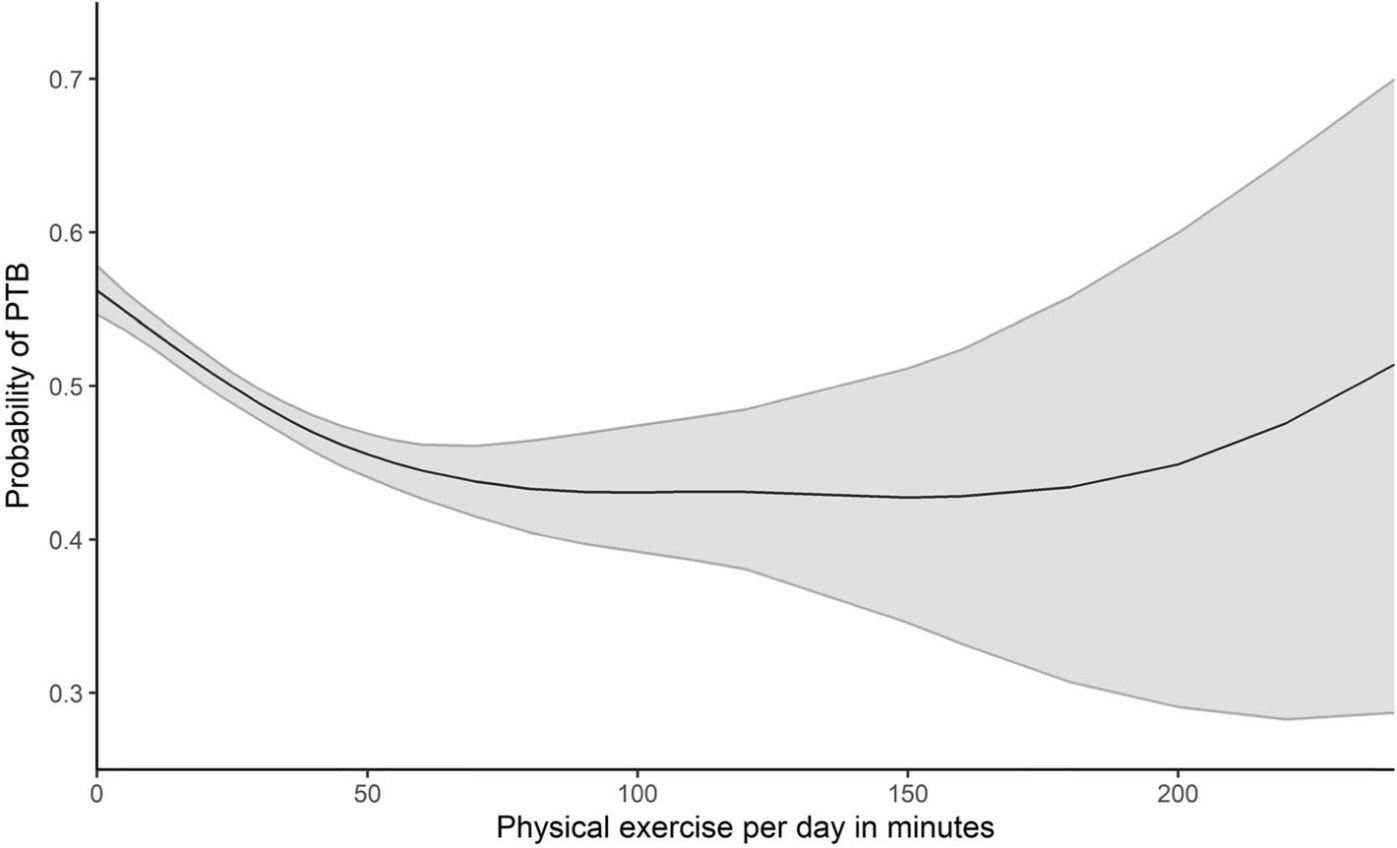
Partial effect estimates of maternal physical exercise per day (in minutes) on the probability of preterm birth based on the Bayesian hierarchical generalized additive model

The parameter estimates of the Bayesian GAMM are summarized in Table 3. The adjusted odds ratios and 95% credible intervals for variables other than physical exercise in minutes are consistent with those in Table 2. Figure 1 demonstrates the relationship between physical activity in minutes (x-axis) and the probability of PTB (y-axis) controlling for other covariates, with 95% credible interval (CI) bands wrapping around the point estimates. Interestingly, there seems to be a “U-shaped” relationship between physical exercise and PTB probability: as physical exercise increased from zero to about 150 min, the probability of experiencing PTB went down. However, once the physical exercise exceeded 150 min per day, the probability of PTB had a moderate increase with more physical exercise. It should be noted that the credible intervals of physical exercise more than 150 min were wide, as there were few participants that exercised more than 150 min per day during pregnancy. Sensitivity analysis using different knots showed a highly similar trend (Supplemental Figure 2).

**Table 3:** Adjusted odds ratios (95% credible intervals) for predictors of preterm birth, physical exercise per day in minutes included as spline

Characteristics	Odds ratio (95% credible interval)
Physical exercise per day in minutes: included as spline	
Maternal age (Reference: 25 to < 30 years)	
< 20	1.70 (1.45, 1.99)
20–24.9	1.17 (1.03, 1.32)
> =30	1.27 (0.98, 1.63)
Education (Reference: less than high school)	
High school or occupational school	0.97 (0.83, 1.15)
College	1.16 (0.99, 1.37)
Graduate school	0.98 (0.73, 1.29)
Income (Reference: middle income 3000–6999 RMB)	
Low income (< 3000 RMB)	1.22 (1.02, 1.46)
High income (> = 7000 RMB)	1.06 (0.93, 1.20)
Family history	2.30 (1.81, 2.94)
Neonatal gender: male	1.28 (1.15, 1.42)
Parity: more than one	0.97 (0.82, 1.13)
BMI prior to pregnancy (Reference: normal weight)	
Underweight	1.02 (0.90, 1.17)
Overweight/Obesity	1.01 (0.81, 1.26)
Weight gain (per one kilogram increase)	0.96 (0.94, 0.97)
Maternal comorbidity	1.71 (1.41, 2.08)
Anemia	0.98 (0.82, 1.18)
Colporrhagia	1.91 (1.70, 2.15)
Indoor air pollution	0.97 (0.85, 1.12)

Maternal comorbidity includes any of the following comorbidities: diabetes, thyroid disease, hepatitis, hypertension, heart disease, renal disease, epilepsy, asthma, depression, anxiety, or others

Similar to the previous models, the effective sample size was at least 3000 for all parameters and the associated Gelman-Rubin statistics were all less than 1.1; the four chains for the Bayesian GAMM were well-mixed without any divergent chains or significant serial-correlation (Supplemental Figure 1B). The evidence indicated that the MCMC algorithms converged and that the estimates were valid and trustworthy.

## Discussion

We found maternal physical exercise 1–2 times, 3–4 times, and over 5 times per week was associated with 20% (aOR = 0.80, 95% CI: 0.68–0.92), 30% (aOR: 0.70, 95% CI: 0.60–0.82), and 32% (aOR = 0.68, 95% CI: 0.59–0.78) lower odds of PTB, respectively, when compared to women who were not involved in any physical activity. In addition, we observed a “U-shaped” relationship between physical exercise in minutes and the probability of PTB in the Bayesian GAMM. The probability of PTB decreased as physical exercise increased from zero to 150 min per day, but began to increase as physical exercise exceeded 150 min per day. Our study supports the previous literature that found a relationship between PTB and physical activity [8, 10, 13–15], with the striking nuance that, while lower levels of physical activity are associated with reduced risk for PTB, higher levels of physical activity are associated with an increased risk for PTB.

Although the underlying mechanism between physical exercise and PTB is not entirely clear, several hypotheses can potentially explain the generally inverse relationship. First, maternal physical exercise can improve the mother’s psychological well-being, lowering the chances of depression during pregnancy and, thereby, preventing PTB [36, 37]. Additionally, insulin sensitivity can be increased with physical exercise and contribute to reducing inflammatory response, which is reported to be a significant risk factor for PTB [9, 11]. Other physiological pathways by which physical exercise helps prevent PTBs, such as exercise improving placental vascularization and reducing oxidative stress and gestational diabetes mellitus, have been suggested by other cohort studies and RCTs [10, 38, 39].

Previous investigations have generally yielded consistent conclusions with our logistic regression results. For example, Juhl et al. reported an adjusted hazard ratio of 0.82 (95% confidence interval: 0.76–0.86) in exercising pregnant women compared with non-exercising pregnant women based on a Danish birth cohort [8]. Guendelman et al. conducted a case-control study based on pregnant women in Southern California and reported an inverse association between moderate exercise in the second trimester and PTB (aOR: 0.90, 95% confidence interval: 0.84–0.96) [13]. Compared to these studies, our study yielded point estimates of greater magnitude (aOR all less than 0.80), which may be explained by Chinese pregnancy culture. In traditional Chinese medicine and culture, pregnancy is a vulnerable period when the mother needs to recuperate and remain sedentary and is often associated with numerous antenatal taboos [40]. It is reported that pregnant women in China have much less physical exercise than pregnant women in Western countries [20]. Therefore, the magnitude of exercise effect among the Chinese population may be higher than that among Western populations. This is supported by another Chinese study on the association between maternal exercise and PTB, which found that adjusted odds ratios for low, medium, and high exercise frequency were all less than 0.70 and all statistically significant [15].

The two models in our study reached similar conclusions that physical exercise is negatively associated with PTB risk. However, the Bayesian GAMM model suggested that the risk of PTB increased as pregnant women engaged in more than 150 min of exercise per day, which was different to the results from the Bayesian logistic regression model. The two predictors were measuring maternal physical activity from different perspectives: the predictor in Bayesian GAMM measured the amount of physical exercise time per day in minutes, while the predictor in Bayesian logistic regression measured the frequency of exercise per week as a categorical variable. The results from the two regression models suggest that pregnant women should engage in frequent physical activity of moderate intensity and length to reduce the risk of PTB but should not exercise for an overwhelming amount of time.

Compared to previous studies, ours has several strengths. First, the participants in this study were recruited from all seven core, urban districts of a large metropolitan city in the same period in China. In this way, unmeasured potential confounders like prenatal care quality and geographic distance to high-quality health care facilities are minimized since there is less variability in these potential confounders among the study population. We also used a Bayesian GAMM to detect the nonlinear relationship between maternal exercise and risk of PTB. Previous investigations have used either a dichotomous or a multi-category variable to measure physical exercise, which could have led to exposure misclassification bias. By contrast, the Bayesian GAMM and quantitative measurement of exercise in this study demonstrated a “U-shaped” relationship between maternal physical exercise and PTB.

Despite these strengths, this study also possesses some limitations. First, our key exposure variable, maternal physical exercise, was self-reported and subject to recall bias in terms of both quantity and quality of exercise. The social desire to be physically active may have led participants to over-report their activity [13]. Second, we did not have detailed physical exercise data for different gestational ages. Third, since this is a case-control study, we are not able to capture all confounders, such as whether PTB was spontaneous or medically induced, spousal support, and physical exercise in different trimesters. Fourth, the participants in this study are primarily young, healthy Chinese women with few who were obese or overweight; therefore, our results and conclusions may not be generalizable to Western population, in which overweight and obesity are major issues in pregnancy. Fifth, controls were matched to any type of preterm birth, but the association between maternal physical exercise and the risk of preterm birth may vary for different types of preterm birth, such as early (< 32 weeks) and late (34–36 weeks) preterm births.

In conclusion, our study adds to the evidence that more maternal physical exercise is associated with lower risk of PTB, but overwhelming physical exercise may increase the risk of PTB. Pregnant women are encouraged to engage in frequent physical exercise of moderate intensity and length. Further study is needed to substantiate the positive association at higher levels of physical exercise and determine the threshold of physical exercise that leads to an increasing risk of PTB. A clearer understanding of the frequency and quality of recommended exercise can help nations around the globe recommend appropriate levels of physical activity for pregnant women.

## Supplementary Information

**Additional file 1: Supplemental Figure 1A.** Trace plots of parameters for the five-category physical exercise variable in the Bayesian hierarchical logistic regression. **Supplemental Figure 1B.** Trace plots of parameters for the splines in the Bayesian generalized additive mixed model. **Supplemental Figure 2.** Sensitivity analysis on partial effect estimates of maternal physical exercise per day (in minutes) on the probability of preterm birth using different knots

## Data Availability

The data set used in this study cannot be made public since it involves private patient identifying information. The datasets generated and/or analyzed during the current study are not publicly available due to the fact that it involves private patient identifying information but may be available from the corresponding author on reasonable request.
