# Supplementary material for: Association between maternal outdoor physical exercise and the risk of preterm birth: a case-control study in Wuhan, China
Source: BMC Pregnancy Childbirth. 2021 Mar 12;21:206. doi: 10.1186/s12884-021-03678-9 (PMC7955628; doi:10.1186/s12884-021-03678-9)

**Supplemental Materials**

**Association between maternal outdoor physical exercise and the risk of preterm birth: a case-control study in Wuhan, China**

Miao Cai^1^, Bin Zhang^2^, Rong Yang^2^, Tongzhang Zheng^3^, Guanghui Dong^4^, Hualiang Lin^1^, Steven E. Rigdon^5^, Hong Xian^5^, Leslie Hinyard^6^, Pamela K. Xaverius^5^, Echu Liu^7^, Thomas E. Burroughs^6^, Daire R. Jansson^5^, Morgan H. LeBaige^5^, Shaoping Yang^2*^, Zhengmin Qian^5*^

1. Department of Epidemiology, School of Public Health, Sun Yat-sen University, Guangzhou, Guangdong, 510080, China
2. Wuhan Children’s Hospital (Wuhan Maternal and Child Healthcare Hospital), Tongji Medical College, Huazhong University of Science & Technology, Wuhan, Hubei, 430015, China
3. Department of Epidemiology Brown School of Public Health, RI 02903, USA
4. Department of Toxicology, School of Public Health, Sun Yat-sen University, Guangzhou, Guangdong, 510080 China
5. Department of Epidemiology and Biostatistics, College for Public Health & Social Justice, Saint Louis University, Saint Louis, MO 63104 USA
6. Center for Health Outcomes Research, Saint Louis University, 3545 Lafayette Avenue, Saint Louis, MO 63104 USA
7. Department of Health Management and Policy, College for Public Health and Social Justice, Saint Louis University, Saint Louis MO 63104 USA

**Emails for correspondence:**

Shaoping Yang, MD, email: [1464502946@qq.com](mailto:1464502946@qq.com)

Zhengmin Qian, MD, PhD,email: [zhengmin.qian@slu.edu](mailto:zhengmin.qian@slu.edu)

| **Table of Contents** | **Page** |
| --- | --- |
| **Supplemental Figure 1A**: Trace plots of parameters for the five-category physical exercise variable in the Bayesian hierarchical logistic regression | 1 |
| **Supplemental Figure 1B**: Trace plots of parameters for the splines in the Bayesian generalized additive mixed model | 1 |
| **Supplemental Figure 2**: Sensitivity analysis on partial effect estimates of maternal physical exercise per day (in minutes) on the probability of preterm birth using different knots. | 2 |

**Supplemental Figure 1A:** Trace plots of parameters for the five-category physical exercise variable in the Bayesian hierarchical logistic regression


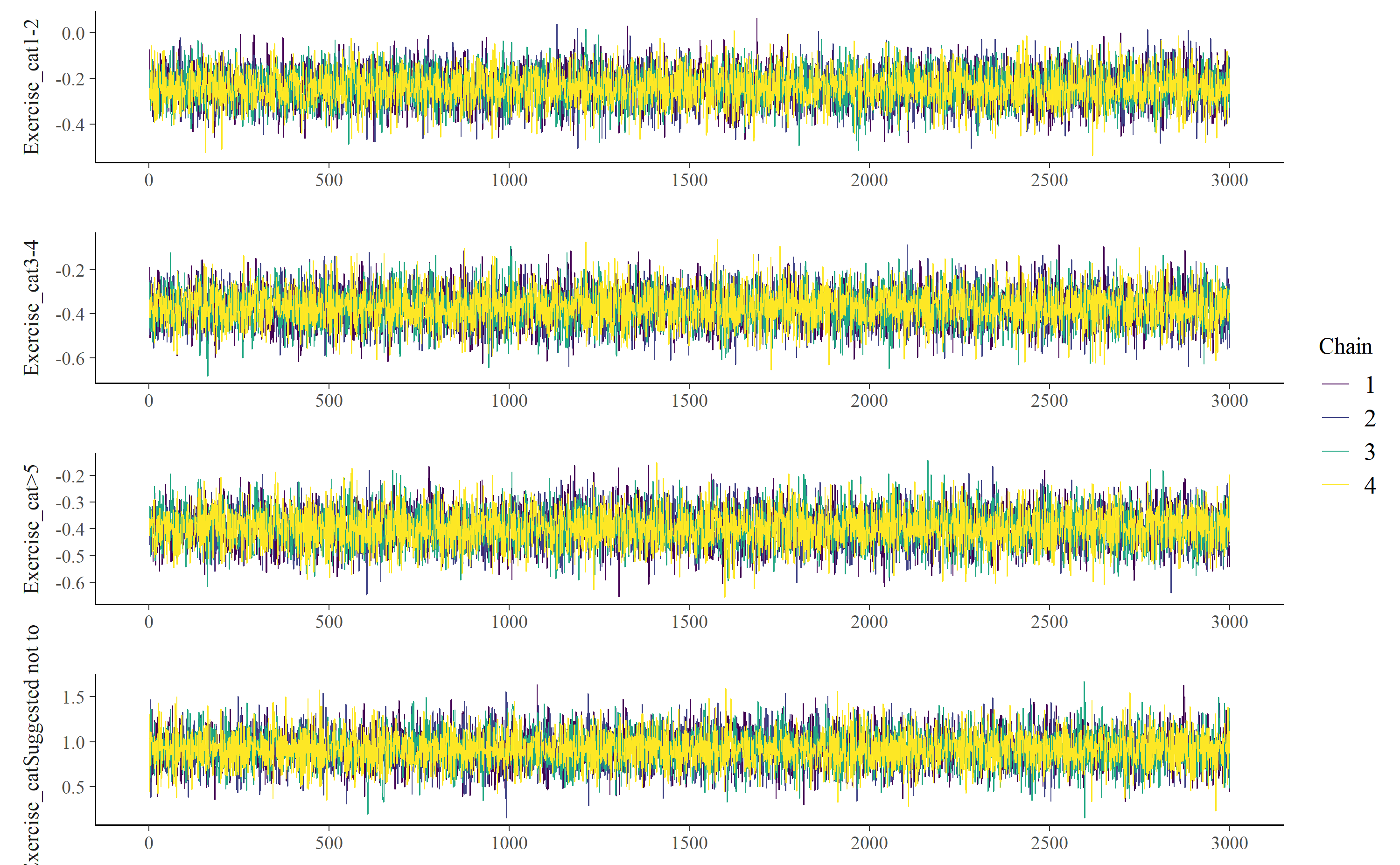


**Supplemental Figure 1B:** Trace plots of parameters for the splines in the Bayesian generalized additive mixed model


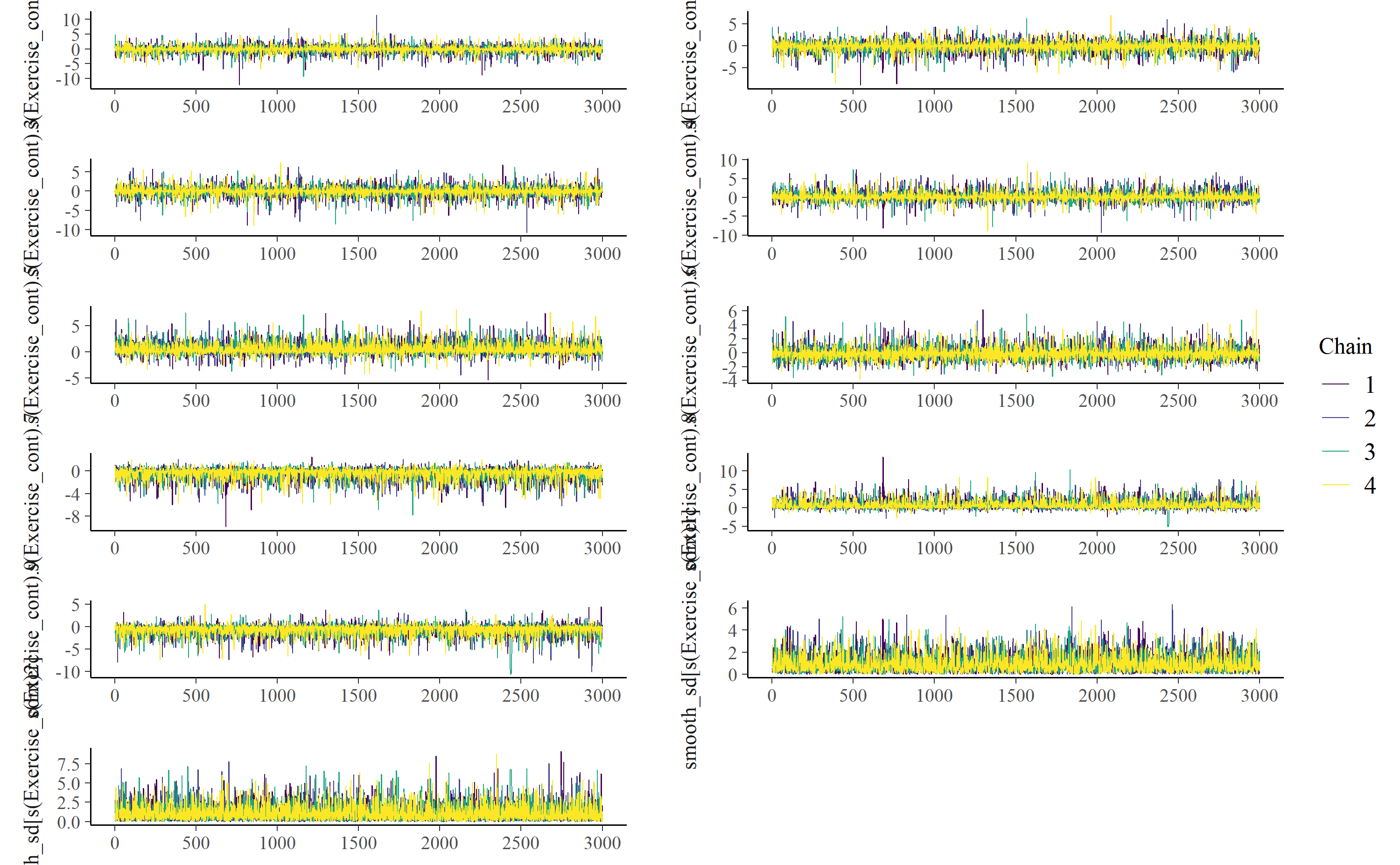


**Supplemental Figure 2**: Sensitivity analysis on partial effect estimates of maternal physical exercise per day (in minutes) on the probability of preterm birth using different knots.


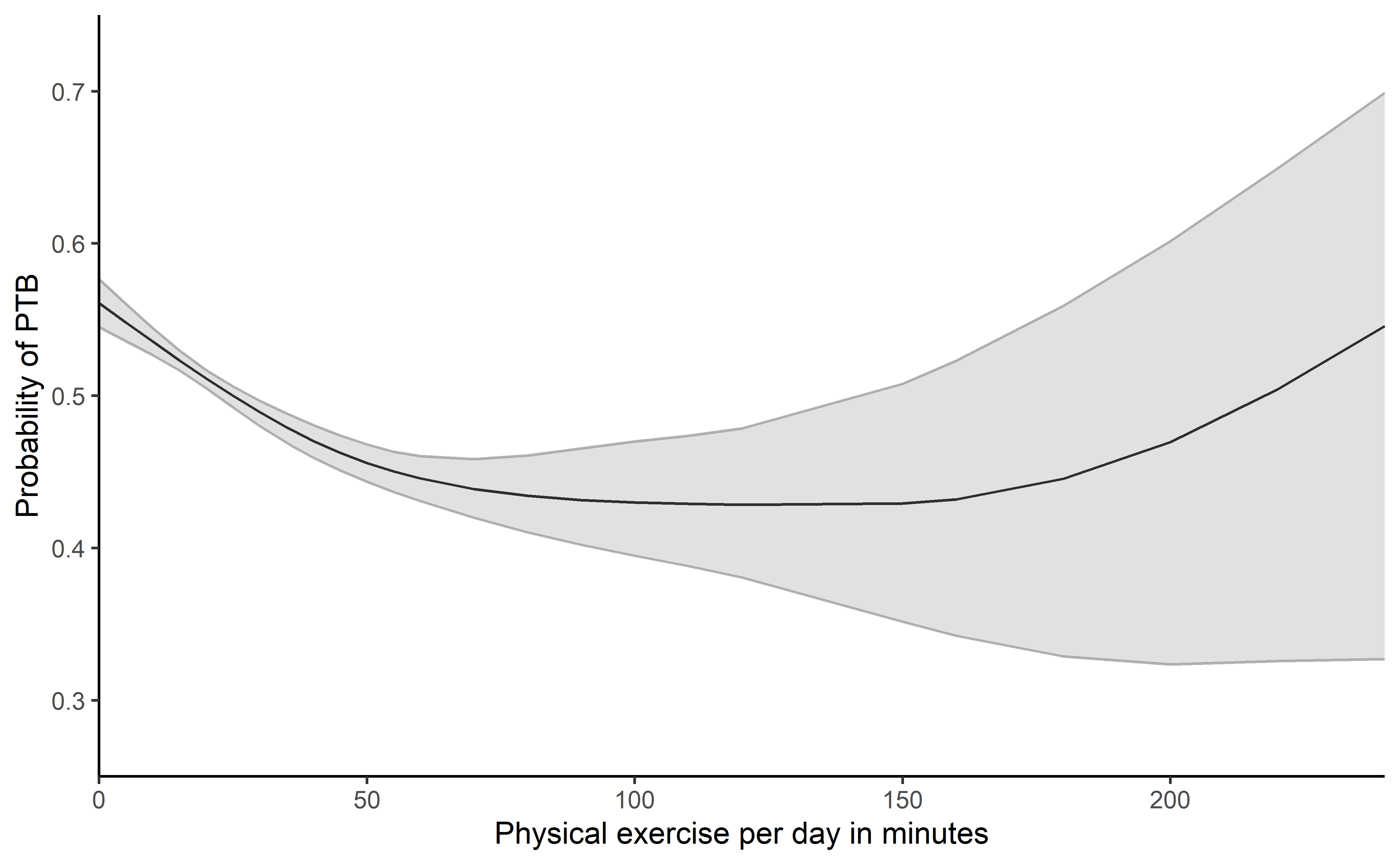

Supplement: Supplementary file 1 — Additional file 1: Supplemental Figure 1A. Trace plots of parameters for the five-category physical exercise variable in the Bayesian hierarchical logistic regression. Supplemental Figure 1B. Trace plots of parameters for the splines in the Bayesian generalized additive mixed model. Supplemental Figure 2. Sensitivity analysis on partial effect estimates of maternal physical exercise per day (in minutes) on the probability of preterm birth using different knots [file 12884_2021_3678_MOESM1_ESM.docx]
